# Focal Choroidal Excavation in a Case of Choroidal Osteoma Associated with Choroidal Neovascularization

**DOI:** 10.18502/jovr.v15i3.7461

**Published:** 2020-07-29

**Authors:** Mahdieh Azimizadeh, Seyedeh Maryam Hosseini, Esmaeil Babaei

**Affiliations:** ^1^Eye Research Center, Mashhad University of Medical Sciences, Mashhad, Iran; ^2^Retina Research Center, Mashhad University of Medical Sciences, Mashhad, Iran; ^3^Eye Research Center, Yazd University of Medical Sciences, Yazd, Iran

**Keywords:** Bevacizumab, Choroidal Excavation, Choroidal Neovascularization, Choroidal Osteoma, Multifocal

## Abstract

**Purpose:**

To report a case of choroidal osteoma associated with reactivation of choroidal neovascularization (CNV) and development of focal choroidal excavation (FCE).

**Case Report:**

A 34-year-old woman with choroidal osteoma complicated by CNV in the right eye for two years presented with deterioration of visual acuity in her right eye. A small retinal hemorrhage accompanied by subretinal fluid (SRF) was seen in the macular area of the right eye. Optical coherence tomography (OCT) showed that the inner retina was intact, and the outer retinal layers had outward displacement. SRF and a wedge-shaped choroidal depression were also seen. This choroidal excavation was not present on previous OCT images. The integrity of the inner retinal layers was maintained, and an optically clear space was present between the neurosensory retina and the retinal pigment epithelium.

**Conclusion:**

Choroidal osteoma can be complicated by CNV and FCE could occur as a consequence. Again, FCE can lead to CNV development. This cascade can deteriorate vision and sometime lead to permanent visual loss.

##  INTRODUCTION

Choroidal osteoma is a rare, ossifying, benign tumor of the choroid. It occurs predominantly in young healthy women in the second and third decades of life and is unilateral in 80% of cases.^[1]^ Although it has been recognized as a benign tumor, it can grow in 51% of patients by 10 years and can be complicated by enlargement, decalcification, and choroidal neovascularization (CNV).^[2]^

**Figure 1:**
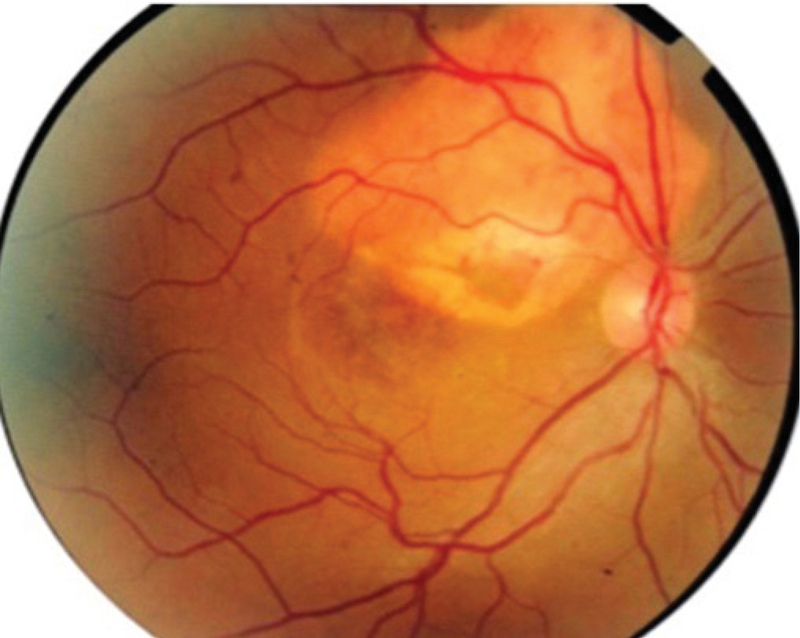
Color fundus photograph of the right eye.

**Figure 2:**
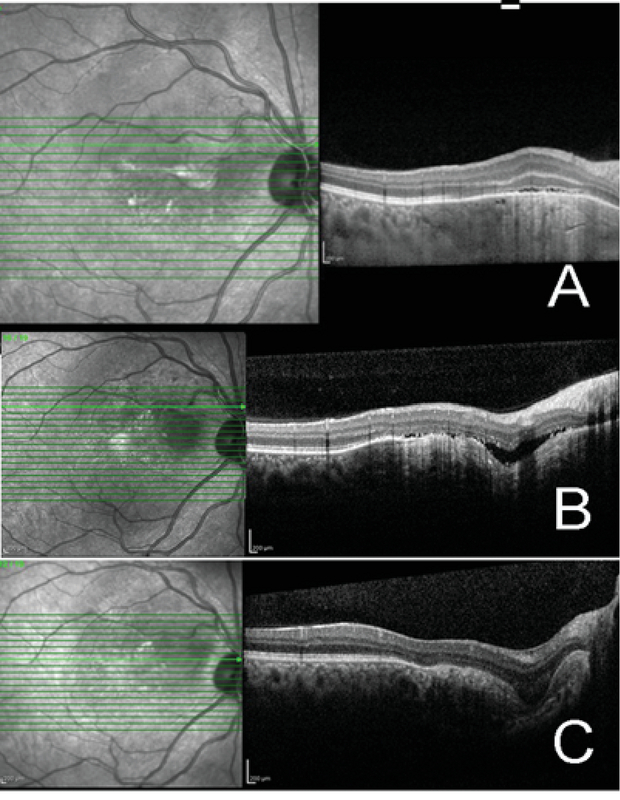
SD- OCT of the right eye: (A) Spectral-domain OCT (SD-OCT) of the right eye performed in 2014 revealed subretinal fluid (SRF) associated with choroidal osteoma complicated by choroidal neovascularization (CNV). (B) OCT of the right eye (2016) clearly shows focal choroidal excavation and SRF due to CNV development. (C) OCT of the right eye clearly shows FCE and complete resolution of SRF after two consecutive intravitreal bevacizumab injections.

**Figure 3:**
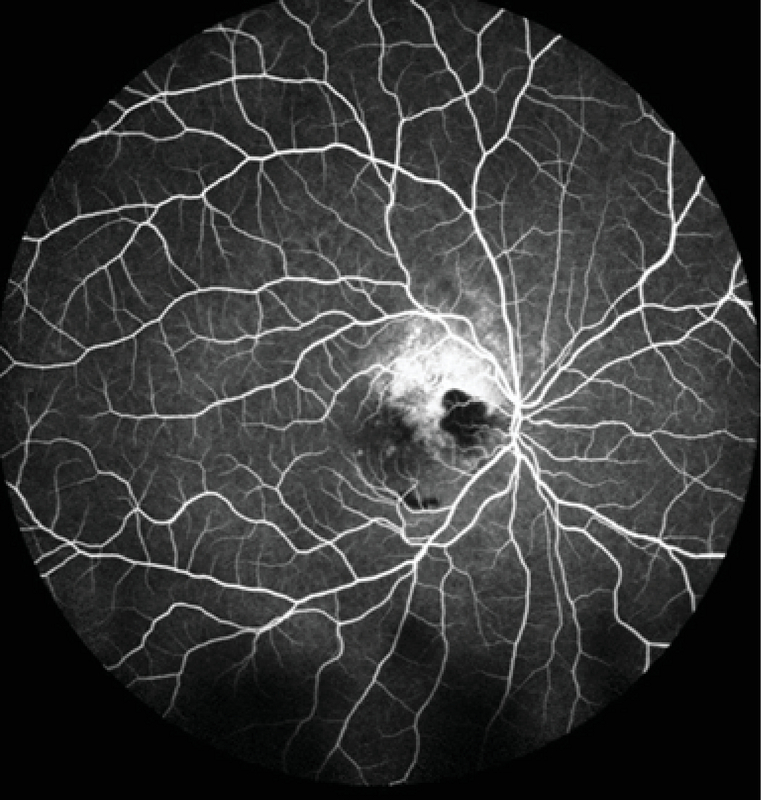
Fluorescein angiography (FAG) showing choroidal neovascularization in the right eye.

**Figure 4:**
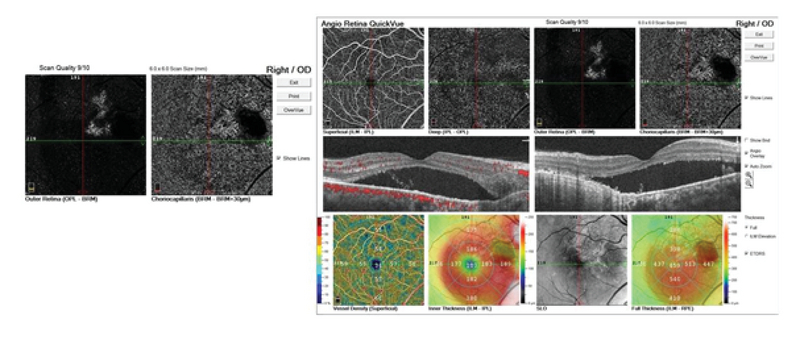
OCTA images of the right eyes illustrate high-flow vessels above the RPE in the outer retina as well as in the choriocapillaris.

Focal choroidal excavation (FCE) refers to focal depression of the choroid that has been recently described thanks to the development of spectral-domain optical coherence tomography (SD-OCT). It may be solitary or occur in association with other chorioretinal disorders such as central serous chorioretinopathy (CSC), CNV, and polypoidal choroidal vasculopathy (PCV).^[3]^ It has also been reported in eyes with choroidal osteoma associated with CNV.^[4,5]^

Here, we report an interesting case involving a 34-year-old woman with bilateral choroidal osteoma complicated by CNV in the right eye, with development of FCE following reactivation of the CNV.

##  CASE REPORT

A 34-year-old healthy woman with bilateral choroidal osteoma complicated by CNV in the right eye for the two years presented with acute deterioration of visual acuity (VA) in the right eye. The findings of multimodal imaging for this case were reported and published in 2015, and there was no FCE in the right eye at that time.^[6]^

Best corrected visual acuity (BCVA) of the right eye had decreased from 20/40 to 20/100, while that of the left eye was 20/20. Fundus examination of both eyes showed a yellow–orange lesion with sharp borders at the level of the choroid (Figure 1). A small retinal hemorrhage accompanied by subretinal fluid (SRF) was seen in the macular area of the right eye. Enhanced depth imaging OCT (EDI-OCT) (Spectralis HRA + OCT, Heidelberg Engineering, Heidelberg, Germany) showed that the inner retina was intact, although the outer retinal layers had outward displacement due to active CNV along with SRF and a wedge-shaped choroidal depression in the right eye. The choroidal excavation involved the retinal pigment epithelium (RPE) and outer retinal layers up to the outer plexiform layer. The integrity of the inner retinal layers was maintained, and an optically clear space was present between the neurosensory retina and RPE. EDI-OCT showed thinning of the choriocapillaris and large choroidal vessels and a sponge-like choroidal lesion (Figure 2). Fluorescein angiography (FAG) demonstrated mild hyperfluorescence in the choroidal osteoma due to window defect, as well as juxtafoveal hyperfluorescence corresponding to active CNV in the right eye (Figure 3), which was better delineated on OCT angiography (Figure 4).

Thus, the diagnosis was reactivation of CNV associated with the development of FCE in the right eye in a known case of choroidal osteoma. The patient received two consecutive intravitreal bevacizumab (Avastin, Genentech Inc., San Francisco, CA) injections, which resulted in improvement of BCVA to 20/40. OCT showed disappearance of SRF and resolution of the disturbances in the outer retinal layers. FCE was more clearly visible and associated with severe choroidal thinning after quiescence of the CNV (Figure 2c). BCVA was maintained in both eyes during a follow-up period of one year.

##  DISCUSSION

We report development of FCE following reactivation of CNV after two years of inactivity in an eye with choroidal osteoma.

Initially, FCE was defined as choroidal excavation in the submacular area without posterior staphyloma, any positive history of trauma, or any chorioretinal disease; however, some chorioretinal disorders such as CSC, PCV, CNV, and choroidal osteoma have been reported in association with FCE.^[7]^ Although the inner retina is commonly intact in eyes with FCE, the outer retinal layers, RPE, choriocapillaris, and choroid can be involved.^[8]^

FCE can be detected by SD-OCT. However, in cases with bilateral involvement or multiple lesions in one eye, foveal SD-OCT which is confined to the posterior pole may not detect all lesions. Therefore, multiple imaging modalities such as fundus photography, fundus autofluorescence, indocyanine green angiography, and FAG are considered helpful.^[9]^

In the present case, FCE developed adjacent to a previous CNV scar and the decalcified part of the tumor as seen on previous OCT images, and it was detected two years after CNV occurrence. Thus, we can conclude that FCE was a consequence of the CNV scar and/or tumor decalcification that resulted in tissue loss, contraction of fibrosis, and outward displacement and excavation of the outer retina and choroid. On the other hand, FCE may have been involved in reactivation of CNV,^[4,5]^ as choroidal excavation has been reported to be associated with the development or reactivation of CNV.^[10]^ There are multiple options for treating CNV in eyes with choroidal osteoma,^[11,12,13,14]^ although the best response has been achieved by intravitreal injection of anti-vascular endothelial growth factor agents such as bevacizumab.^[15]^

This is a documented development of FCE in an eye with CNV associated with choroidal osteoma. FCE could occur as a consequence of CNV, tumor decalcification, or both. Close follow-up and prompt treatment are necessary to achieve the best results.

##  Financial Support and Sponsorship

Nil.

##  Conflicts of Interest

There are no conflicts of interest.

## References

[1] Erol MK, Coban DT, Ceran BB, Bulut M. Enhanced depth imaging optical coherence tomography and fundus autofluorescence findings in bilateral choroidal osteoma: a case report. *Arq Bras Oftalmol* 2013; 76:189-91. 10.1590/s0004-27492013000300012 23929082

[2] Shields CL, Perez B, Materin MA, Mehta S, Shields JA. Optical coherence tomography of choroidal osteoma in 22 cases: evidence for photoreceptor atrophy over the decalcified portion of the tumor. *Ophthalmology* 2007; 114:e53-8 10.1016/j.ophtha.2007.07.037 17884171

[3] Cebeci Z, Bayraktar Ş, Oray M, Kır N. Focal choroidal excavation. *Turk J Ophthalmol* 2016; 46:296. 10.4274/tjo.24445 PMC5177789 28050329

[4] Pierro L, Marchese A, Gagliardi M, Introini U, Parodi MB, Casalino G, et al. Choroidal excavation in choroidal osteoma complicated by choroidal neovascularization. *Eye (Lond)*. 2017;31:1740-1743. 10.1038/eye.2017.136 PMC5733291 28731055

[5] Wakabayashi Y, Nishimura A, Higashide T, Ijiri S, Sugiyama K. Unilateral choroidal excavation in the macula detected by spectral-domain optical coherence tomography. *Acta Ophthalmol* 2010;88:e87-91. 10.1111/j.1755-3768.2010.01895.x 20546234

[6] MirNaghi M, Nasser S, SeyedehMaryam H, Ali ST. Bilateral multifocal choroidal osteoma with cho-roidal neovascularization. *Case Rep Ophthalmol Med* 2015;2015:346415. 10.1155/2015/346415 PMC4684841 26770855

[7] Navarro VC, Hernández JM, Palop CN, Fort PP, Taulet EC. Focal choroidal excavation: Clinical findings and complications. *Arch Soc Esp Oftalmol* 2016;91:34-9. 10.1016/j.oftal.2015.10.004 26652731

[8] Liu GH, Lin B, Sun XQ, He ZF, Li JR, Zhou R, et al. Focal choroidal excavation: a preliminary interpreta-tion based on clinic and review. *Int J Ophthalmol* 2015;8:513-21. 10.3980/j.issn.2222-3959.2015.03.14 PMC4458655 26086000

[9] Chung H, Byeon SH, Freund KB. Focal choroidal excavation and its association with pachychoroid spectrum disorders. *Retina* 2017;37:199-221. 10.1097/IAE.0000000000001345 27749784

[10] Xu H, Zeng F, Shi D, Sun X, Chen X, Bai Y. Focal choroidal excavation complicated by choroidal neovascularization. *Ophthalmology* 2014;121:246-250. 10.1016/j.ophtha.2013.08.014 24095605

[11] Mansour AM, Arevalo JF, Al Kahtani E, Zegarra H, Abboud E, Anand R, et al. Role of intravitreal antivascular endothelial growth factor injections for choroidal neovascularization due to choroidal osteoma. *J Ophthalmol* 2014;2014:210458. 10.1155/2014/210458 PMC4132478 25147732

[12] Shields CL, Sun H, Demirci H, Shields JA. Factors predictive of tumor growth, tumor decalcification, choroidal neovascularization, and visual outcome in 74 eyes with choroidal osteoma. *Arch Ophthalmol* 2005;123:1658-66. 10.1001/archopht.123.12.1658 16344436

[13] Browning D. J, Choroidal osteoma: observations from a community setting, *Ophthalmology* 2003;110:1327-34. 10.1016/S0161-6420(03)00458-5 12867386

[14] Shields CL, Perez B, Materin MA, Mehta S, Shields JA. Optical coherence tomography of choroidal osteoma in 22 cases: evidence for photoreceptor atrophy over the decalcified portion of the tumor. *Ophthalmology* 2007;114:e53-8. 10.1016/j.ophtha.2007.07.037 17884171

[15] Ahmadieh H, Vafi N. Dramatic response of choroidal neovascularization associated with choroidal osteoma to the intravitreal injection of bevacizumab (Avastin). *Graefes Arch Clin Exp Ophthalmol* 2007;245:1731-3. 10.1007/s00417-007-0636-z 17653753

